# Serum albumin and white matter hyperintensities

**DOI:** 10.1038/s41398-024-02953-5

**Published:** 2024-06-01

**Authors:** Natalie M. Zahr, Adolf Pfefferbaum

**Affiliations:** 1 Department of Psychiatry & Behavioral Sciences, Stanford University School of Medicine, Stanford, CA USA; 2 Neuroscience Program, SRI International, Menlo Park, CA USA

**Keywords:** Predictive markers, Learning and memory

## Abstract

People living with HIV and those diagnosed with alcohol use disorders (AUD) relative to healthy individuals commonly have low levels of serum albumin, substantiated as an independent predictor of cardiovascular events. White matter hyperintensities (WMH)—a neuroimaging feature of cerebral small vessel disease—are also related to cardiovascular disease. Despite consensus regarding associations between high levels of urine albumin and WMH prevalence, and low serum albumin levels and impaired cognitive functioning, relations between serum albumin and WMH burdens have rarely been evaluated. Here, a sample including 160 individuals with AUD, 142 living with HIV, and 102 healthy controls was used to test the hypothesis that serum albumin would be inversely related to WMH volumes and directly related to cognitive performance in the two diagnostic groups. Although serum albumin and periventricular WMH volumes showed an inverse relationship in both AUD and HIV groups, this relationship persisted only in the HIV group after consideration of traditional cardiovascular (i.e., age, sex, body mass index (BMI), nicotine use, hypertension, diabetes), study-relevant (i.e., race, socioeconomic status, hepatitis C virus status), and disease-specific (i.e., CD4 nadir, HIV viral load, HIV duration) factors. Further, serum albumin contributed more significantly than periventricular WMH volume to variance in performance on a verbal learning and memory composite score in the HIV group only. Relations in both HIV and AUD groups between albumin and hematological red blood cell markers (e.g., hemoglobin, hematocrit) suggest that in this sample, serum albumin reflects hematological abnormalities. Albumin, a simple serum biomarker available in most clinical settings, may therefore help identify periventricular WMH burden and performance levels in specific cognitive domains in people living with HIV. Whether serum albumin contributes mechanistically to periventricular WMH in HIV will require additional investigation.

## Introduction

People living with HIV relative to healthy control individuals commonly have low serum albumin levels that are prognostic for disease severity and rate of progression [1–3]. In individuals diagnosed with alcohol use disorders (AUD), low serum albumin levels are observed [4, 5] generally in the context of liver disease [6–8]. Additionally, individuals with HIV and AUD comorbidity have high rates of hepatitis C virus (HCV) infection [9–11], which can independently lower serum albumin [12–17].

Hypoalbuminemia (i.e., low serum albumin) occurs in liver cirrhosis, kidney disease, and malnutrition among other diagnoses due to mechanisms such as decreased liver synthesis, increased urinary excretion, or increased catabolism [18–21]. Low serum albumin levels can predict cardiovascular incidents (e.g., coronary artery disease, myocardial infarctions, heart failure, arrhythmias) and stroke after consideration of traditional risk factors such as body mass index (BMI), nicotine use, diabetes, and hypertension [22–35]. Low albumin may promote cardiovascular disease via decreased intravascular oncotic pressure, or mechanisms involving increased oxidative stress (i.e., decreased scavenging) or inflammation [36–40].

Cardiovascular disease plays a key role in the etiology of brain white matter hyperintensities (WMH) [41, 42]. Individuals infected with HIV [43–45] or diagnosed with AUD [46, 47] carry greater liability for cerebrovascular events, including WMH prevalence, than the general population [43, 48–50]. Indeed, WMH volumes enlarge at an accelerated rate in HIV relative to healthy controls [49, 51].

Since 2007, several studies have demonstrated relations between high urine albumin (i.e., albuminuria) and greater WMH prevalence independent of established risk factors (i.e., age, sex, hypertension, diabetes, nicotine use) [52] in both healthy and clinical populations [e.g., 1253 hypertensive participants, Mayo Clinic, MN, US [53]; 285 hypertensive participants, Nagoya, Japan [54]; 975 hypertensive participants, Barcelona, Spain [55]; 1215 healthy older (>60 years) adults, Seoul, Korea [56]; 2,671 healthy older (~75 years) adults, Reykjavik, Iceland [57]; 1,214 community dwelling adults, Fukuoka, Japan [58]; 112 at risk elderly (~68 years), Exeter, UK [59]; 5,324 patients taking oral antithrombotic agents across multiple sites in Japan [60]]. Surprisingly, despite this reproducible relationship between albuminuria and WMH prevalence and accruing evidence for associations between low serum albumin levels and cognitive impairment in a variety of clinical cohorts [e.g., 1,511 patients with heart failure, albumin<3.5 g/dL, multiple sites, Italy [61]; 1,284 adults ≥55 years, albumin=4.4±0.3 g/dL, Amsterdam, Netherlands [62]; 2,550 Chinese adults ≥55 years, albumin<4.0 g/dL, Singapore [63]; 433 hip fracture patients ≥65 years, albumin=3.51±0.47 g/dL, Israel [64]; 1,752 adults ≥65 years, albumin=3.9±0.4 g/dL, multiple sites, England [65]; 191 patients with Parkinson’s Disease, albumin levels unspecified, Pennsylvania, U.S. [66]; 274 patients with acute heart failure, albumin=3.4±0.4 g/dL, Osaka, Japan [67]], the correlation between serum albumin and WHM occurrence has rarely been explored. In 46 patients with systemic lupus erythematosus, low serum albumin (i.e., albumin=3.8±1.3 g/dL) was associated with larger WMH lesion volume [68]. By contrast, a study of 396 older (>55 years) healthy adults in Korea showed that low serum albumin was associated with high cerebral β amyloid reactivity but not with WMH volume [69].

Here, cross-sectional data comprising WMH volumes matched to clinical laboratory measures from 160 individuals with AUD, 142 infected with HIV, and 102 healthy controls were evaluated to test the hypothesis that low serum albumin levels would be associated with larger WMH volumes and worse cognitive performance in both diagnostic groups.

## Methods

### Participants

Cross-sectional neuroimaging and clinical laboratory data from 3 study groups (102 control, 160 AUD, 142 HIV) were extracted from a longitudinal dataset [51] drawn from published studies [51, 70, 71]. All but 18 of the 404 cases were from the first visit in longitudinal study; for these 18 cases, matching time points for blood and imaging markers were only available for the 2^nd^ visit. Participants were recruited from local alcohol and drug recovery centers, HIV clinics, postcard mailings, recruitment flyers, and word of mouth. After obtaining written informed consent for study participation, approved by the SRI International and Stanford University School of Medicine Institutional Review Boards, volunteers underwent a Structured Clinical Interview for Diagnostic and Statistical Manual (DSM)-IV and DSM-5 Disorders (SCID) [72], structured health questionnaires, and a semi-structured timeline follow-back interview to quantify lifetime alcohol consumption [73].

Upon initial assessment, volunteers were excluded if they had a significant history of medical (e.g., liver cirrhosis, epilepsy, stroke, multiple sclerosis, uncontrolled diabetes, or loss of consciousness > 30 min), neurological (e.g., Parkinson’s disease), or psychiatric (e.g., schizophrenia, bipolar disorder) disorders other than an AUD (DSM-5). Other exclusionary criteria were substance dependence (other than alcohol for the AUD group) within the past 3 months or any other DSM disorder (for all groups). All participants also completed screening to ensure MRI safety and a breathalyzer test for recent alcohol consumption. Socioeconomic status (SES) was derived from the *Four-Factor Index of Social Status*, which considers education and occupation level and wherein a lower score reflects higher status [74]. Scores on the Alcohol Use Disorders Identification Test (AUDIT), developed by the World Health Organization as a self-report screening test to identify severity of AUD and provide an overall measure of hazardous drinking, were totaled (higher scores represent more intense drinking) [75–77]. Sitting blood pressure was collected twice. The mean of each of the two diastolic and systolic measures was used. As in other studies, the diagnostic groups relative to the healthy control group were less well-educated, had worse SES, and were more likely to include men, Black individuals, nicotine use, and HCV infection (Table 1) [70, 71, 78, 79].

**Table 1:** Demographic Characteristics by Diagnostic Group: mean ± standard deviation or frequency count.

	Control (*n* = 102)	AUD (*n* = 160)	HIV (*n* = 142)	χ2 or F	*p*-value*
**N (women/men)**	48/54	44/116	44/98	11.4	**0.0034**
**Age (years)**	53.3 ± 15.3	50.8 ± 10.7	52.5 ± 9.2	1.7	0.1935
**Self-Defined Race (White/AA/other**^**a**^**)**	57/20/25	71/62/27	45/70/27	24.2	**<0.0001**
**Handedness (Right/Left)**	90/12	137/23	119/23	4.7	0.3213
**Body Mass Index (BMI)**	26.1 ± 4.3	27.3 ± 4.4	26.2 ± 4.2	3.5	0.0332
**Education (years)**	16.1 ± 2.4	13.4 ± 2.5	13.3 ± 2.4	49.0	**<0.0001**
**Socioeconomic Status**^**b**^	25.5 ± 12.5	41.9 ± 15.4	41.3 ± 13.9	57.6	**<0.0001**
**Smoker (never/past or current)**	93/9	53/107	63 / 79	88.8	**<0.0001**
**Hypertensive**^**c**^ **(yes/no)**	18/84	42/118	26 / 116	3.9	0.1416
**Diabetic**^**d**^ **(yes/no)**	3/99	9/151	10 / 132	2.0	0.3761
**AUD diagnoses (yes/no)**	0/102	160/0	74 / 68	—	—
**AUD duration**	—	25.4 ± 11.4	27.8 ± 11.9	2.2	0.1379
**Lifetime alcohol consumption**	40.7 ± 68.8	1274.2 ± 992.6	524.5 ± 763.1	148.5	**<0.0001**
**AUDIT**^**e**^ **scores**	1.9 ± 1.6	20.4 ± 11.8	7.1 ± 9.3	187.0	**<0.0001**
**Days since last drink**	678.9 ± 2895.8	200.7 ± 541.0	253.6 ± 973.4	1.4	0.2434
**HIV duration (years)**	—	—	17.4 ± 8.4	—	—
**CD4 cell count (100/mm**^**3**^**)**	—	—	580.5 ± 296.0	—	—
**CD4 cell-count nadir (100/mm**^**3**^**)**	—	—	187.8 ± 153.1	—	—
**Viral load (log copies/mL)**	—	—	2.1 ± 1.1	—	—
**AIDS-defining**^**f**^ **event (yes/no)**	—	—	14/128	—	—
**ART**^**g**^ **(yes/no)**	—	—	125/17	—	—
**Hepatitis C Virus (HCV**±**)**	1/101	34/126	54/88	47.5	**<0.0001**
**Treatment for HCV**^**h**^ **(yes/no)**	0/102	9/25	9/45	6.5	0.0392
**albumin (g/dL)**	4.46 ± 0.28	4.44 ± 0.31	4.28 ± 0.41	10.1	**<0.0001**
**albumin** < **3.5** **g/dL**	0	0	6	11.2	**0.0036**

*3-group (control, *AUD* alcohol use disorder, *HIV* human immunodeficiency virus) comparisons used Welch’s test for continuous variables or χ^2^ for categorical variables. ^a^other Native American, Asian, Islander; ^b^lower Score=higher status; ^c^Hypertensive systolic ≥ 140 or diastolic ≥ 190; ^d^self-report of diabetes; ^e^*AUDIT* Alcohol Use Disorders Identification Test; ^f^AIDS-defining illness or CD4 prior nadir <200cells/μl; ^g^*ART* active retroviral therapy; ^h^self-report of HCV treatment; Bold signficant at Bonferroni-corrected *p*-value ≤ 0.005 (for 10 demographic variables).

### Neuroimaging acquisition and analysis

#### Protocols and parameters

Scanning was conducted at SRI International on a GE Discovery MR750 system (Waukesha, WI, U.S.A.) with ASSET for parallel and accelerated imaging on an 8-channel head coil. Detection and localization of WMH used three magnetic resonance imaging (MRI) acquisition protocols: T1-weighted (T1-w) MRI for anatomical localization: 3D axial IR-Prep (inversion prepared) SPGR (SPoiled Gradient Recalled); repetition time (TR) = 6.5 ms, echo time (TE) = 1.54 ms, thickness (thick)=1.25 mm, locations (loc) =124, skip=0); T2-weighted (T2-w) MRI merged with T1-w data for skull stripping: 3D isotropic FSE (Fast Spin Echo; GE Name=CUBE), TR = 2500 ms, effective TE = 99 ms, echo train length (ETL) = 100 ms, thick=1 mm, loc=150, FOV = 256 mm, xy_matrix=256×256, Resolution=1 × 1 × 1 mm; and FLAIR (FLuid-Attenuated Inversion Recovery) imaging for estimates of WMH volumes: 2D axial, TR = 9000 ms, TE = 82.5 ms, inversion time (TI) = 2200 ms, thick=2.5 mm, loc=65.

#### MRI structural analysis

Preprocessing of T1-weighted SPGR data involved noise removal [80] and brain mask segmentation using FSL BET [81], AFNI 3dSkullStrip [82], and Robust Brain Extraction (ROBEX) [83] generating 3 brain masks. In parallel, noise-corrected, T1-weighted images were corrected for field inhomogeneity via N4ITK [84], brain masks were segmented [85], and the resulting segmented brain masks were reduced to one using majority voting [86]. Brain tissue segmentation (gray matter, white matter, and cerebrospinal fluid) of the skull-stripped T1-weighted images was generated via Atropos [84]. Parcellated maps of tissue used the parc116 atlas to define cortical (gray matter) and subcortical (gray and white matter) volumes summed for bilateral hemispheres.

#### WMH quantification

WMH analysis was accomplished with the “UBO Detector,” acluster-based, fully automated pipeline for extracting and calculating WMHs on a voxel basis [87]. This procedure yielded voxel maps for 2 WMH volumes: periventricular and deep. The pipeline required that FLAIR and T1-w data be warped into MNI space prior to non-rigid transformation into standard SRI atlas space. This was necessary for accurate placement of anatomical locations to enable comparisons across individuals and across imaging modalities on a voxel-wise basis without the need for further correction for differences in intracranial volume.

### Blood sample collection

Blood samples were collected for analysis by Quest Diagnostics for complete blood count (CBC) (test code: 6399, CPT: 85025), comprehensive metabolic panel (test code: 10231, CPT code: 80053), and HIV and HCV screening with RNA quantification for seropositive individuals. CBC required whole blood collected in EDTA tubes; remaining tests used serum separator tubes (SST) tubes. The Quest Diagnostics reference range for serum albumin is 3.6–5.1 g/dL; levels ≤3.5 g/dL were considered below range [88].

To evaluate the significance of serum albumin, its relations with other blood biomarkers were considered. Relations between serum albumin and levels of aspartate aminotransferase (AST), alanine aminotransferase (ALT), *γ*-glutamyl transferase (GGT), and alkaline phosphatase were used to evaluate liver disease; with eGFR and creatinine levels for kidney disease; with levels of vitamins B9 (folate) and B12 (cobalamin) for malnutrition; and with TNFα and IP10 levels for inflammation [for details on methods for cytokine measures see: [78, 89].

### Cognitive composite scores

Cognitive composite scores matched to date of blood draw for each participant were extracted from an in-house laboratory release as described [51, 90]. Briefly, composites cognitive scores were created by averaging age-, education-, and sex-corrected Z-scores on performance on neuropsychological tests. Composites scores comprised tests of executive functioning, attention and working memory, visual and verbal learning, and visual and verbal memory as listed.

executive functioning

Trails B time *or* Color-trails time 2 +

Digit symbol raw score at 90 s or Symbol digit raw score at 90 s+

Phonological fluency (sum of unique “F” + “A” + “S” words)

attention and working memory

Trails A time *or* Color-trails time1 +

Wechsler Memory Scale-Revised (WMS-R) digits forward raw score total +

WMS-R digits backwards raw score total +

WMS-R block tapping forward total +

WMS-R block tapping backward total

visual and verbal learning

Rey-Osterrieth complex figure immediate raw score +

WMS-R logical memory immediate total

visual and verbal memory

Rey-Osterrieth complex figure delay raw score +

WMS-R logical memory delay total

### Statistics

Statistics were performed using JMP® Pro 16.0.0 (SAS Institute Inc., Cary, NC, 1989-2021). For demographics, χ^2^ was used on categorical variables, Welch’s test for unequal variances was used on continuous variables. Significance required Bonferroni-corrected p-values as indicated in text or tables. Initial stepwise regression models for periventricular or deep WMH volumes selected from among 9 traditional cardiovascular risk variables [age, sex (male/female), race (black/white/other), BMI, SES, HCV status (positive/negative), nicotine use (never/past or current, self-report), hypertension (yes/no, yes = systolic ≥ 140 or diastolic ≥ 90), diabetes (yes/no, self-report)], serum albumin levels, and 3 disease specific markers per diagnoses (for AUD: total AUDIT score, lifetime alcohol consumed, AUD duration; for HIV: CD4 nadir, HIV viral load, HIV duration). These were followed by multiple regression analyses including only the variables selected by stepwise regressions. Performance in 4 cognitive domains also used multiple regressions. Factors were identified as significant contributors to variance in multiple regression models if they passed FDR (false discovery rate)-corrected logworth contribution to total variance. JMP uses Benjamini-Hochberg FDR p-value adjustment (defined as -log10[p-value]) to standardize p-values to permit comparison across multiple tests [91]. To confirm a priori selected variables were related, principal component analysis (PCA) on correlations were used to cluster variables. JMP iteratively splits clusters of variables and reassigns variables to clusters until no more splits are possible based on an algorithm developed by SAS [92].

## Results

**Fig. 1:**
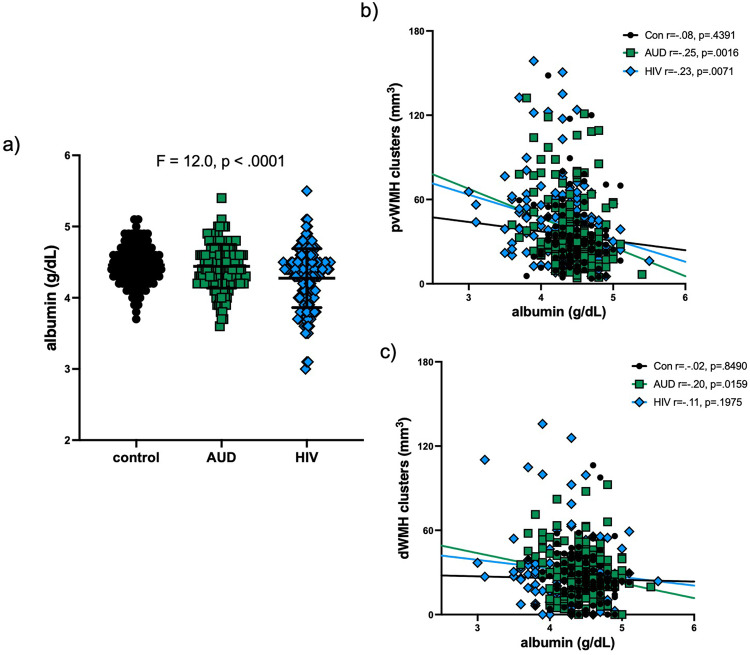
Serum albumin and white matter hyperintensity (WMH) volumes. **a** Serum albumin levels in individuals with Alcohol Use Disorders (AUD), infected with HIV, and healthy controls. **b** Serum albumin levels relative to periventricular (pv)WMH volumes in the 3 study groups. **c** Serum albumin levels relative to deep WMH (dWMH) volumes in the 3 study groups.

Serum albumin levels were low in the HIV relative to the AUD and healthy control groups (Fig. 1a). Collapsed across diagnosis groups, infection with HCV was also associated with low serum albumin levels (F = 26.0, *p* < 0.0001). The AUD (*r* = -0.25, *p* = 0.0016) and HIV (*r* = -0.23, *p* = 0.0071) groups showed an inverse relationship between serum albumin and periventricular WMH volume (Fig. 1b). Only the AUD group showed an inverse relationship between serum albumin and deep WMH volume (*r* = -0.20, *p* = 0.0159; Bonferroni-correction for 3 group comparisons, *p*-value = 0.05/3 = 0.0167; Fig. 1c). The HCV group (*n* = 88) did not show significant relations between serum albumin and periventricular (*r* = -0.12, *p* = 0.2728) or deep (*r* = -0.18, *p* = 0.0927) WMH volumes.

**Fig. 2:**
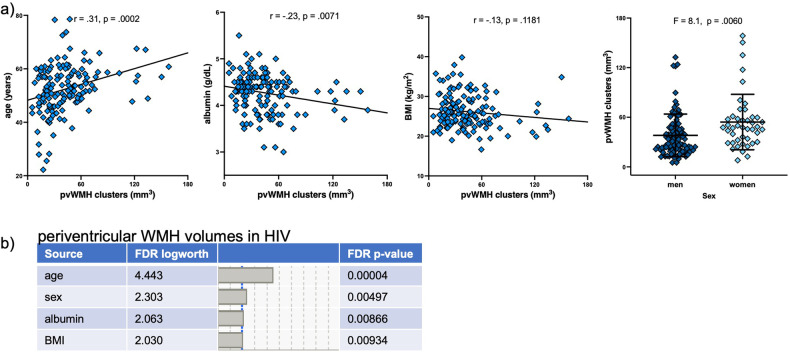
Variables relevant to white matter hyperintensity (WHM) volumes in HIV. **a** Variables in the HIV group contributing significantly to periventricular (pv) WMH volumes included age, sex, serum albumin, and body mass index (BMI). **b** List of these variables by their relative false discovery rate (FDR) contribution to pvWMH volume in the HIV group.

For periventricular WMH volume in the HIV group, the stepwise regression selected 4 (age, sex, BMI, and serum albumin, Fig. 2a) from among 13 (age, sex, race, SES, BMI, HCV status, nicotine use, presence of hypertension or diabetes, serum albumin levels, CD4 nadir, HIV viral load, years with HIV) variables. The follow-up multiple regression model in the HIV group including these 4 factors was significant (F_4,141_ = 11.0, *p* < 0.0001), explained 24.3% of the variance in periventricular WMH volumes, and was driven by age (p = 0.00004) with similar FDR significant contributions from sex (p = 0.005), serum albumin (*p* = 0.009), and BMI (p = 0.009, Fig. 2b).

**Fig. 3:**
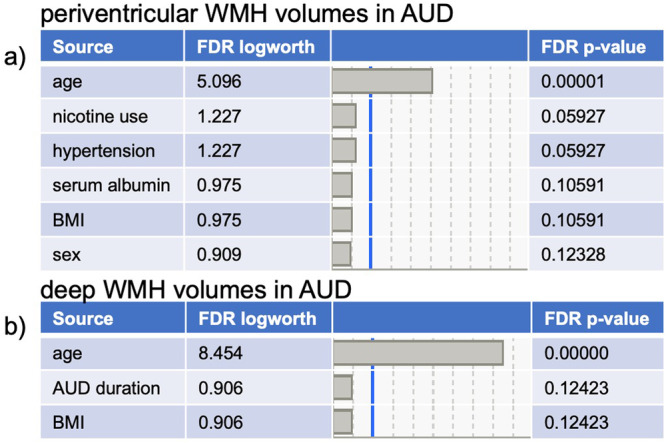
Variables relevant to white matter hyperintensity (WHM) volumes in AUD. **a** List of variables contributing with false discovery rate (FDR) significance to periventricular (pv) WMH volumes in the Alcohol Use Disorders (AUD) group. **b** List of variables contributing with FDR significance to deep WMH (dWMH) volumes in the AUD group.

In the AUD group, the stepwise regression identified 6 (i.e., age, sex, BMI, nicotine use, hypertension, and serum albumin) of 13 (age, sex, race, SES, BMI, HCV status, nicotine use, presence of hypertension or diabetes, serum albumin levels, total AUDIT score, lifetime alcohol consumed, years with AUD) variables as relevant to periventricular WMH volumes. The follow-up multiple regression in the AUD group including these 6 variables was significant (F_6,159_ = 9.1, *p* < 0.0001) and explained 26.4% of the variance in periventricular WMH volume; the only variable that contributed with FDR significance to the model, however, was age (*p* < 0.00001, Fig. 3a). For deep WMH volumes in the AUD group, the stepwise regression selected 3 (i.e., age, BMI, AUD duration) of the 13 variables The follow-up multiple regression in the AUD group was significant (F_3,157_ = 16.9, *p* < 0.0001) and explained 24.7% of the variance in deep WMH volumes; again, the only variable that contributed with FDR significance to the model was age (*p* < 0.00001, Fig. 3b).

**Fig. 4:**
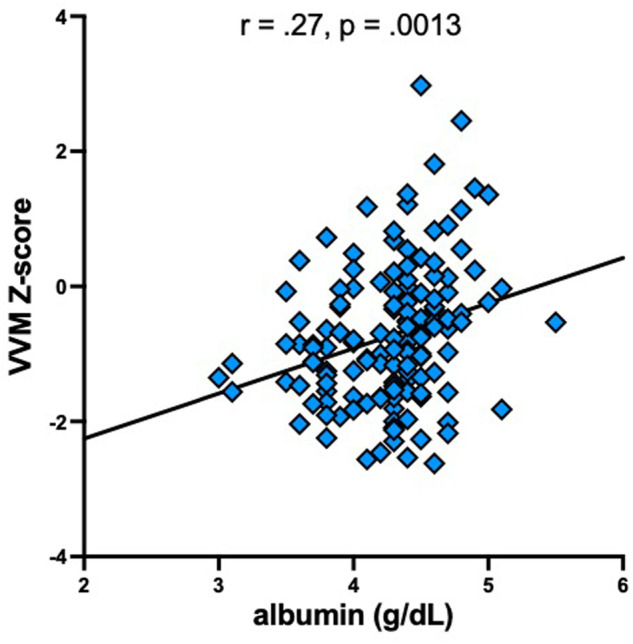
Relations in the HIV group between serum albumin levels and the visual and verbal memory (VVM) cognitive composite score.

Next, the contributions of serum albumin levels and periventricular WMH volumes to cognitive performance in 4 domains in the HIV group were evaluated. Serum albumin contributed with more FDR-weighted significance than WMH volume to performance in the visual and verbal memory domain (Table 2, Fig. 4). Similar analyses in the AUD group revealed comparable patterns not reaching significance (Table S1).

**Table 2:** Results of multiple regression models* for cognitive perfomance in the HIV group.

	model		R^2^	principal source			secondary source		
	F_2,141_	p		source	FDR lw	FDR p	source	FDR lw	FDR p
Executive functioning	6.6	0.0018	8.9	pvWMH	1.5	0.03	albumin	1.4	0.04
Attention and working memory	1.5	0.2216	2.2	pvWMH	1.1	0.09	albumin	0.03	0.56
Visual and verbal learning	6.1	0.0029	8.2	albumin	1.8	0.02	pvWMH	0.9	0.12
Visual and verbal memory	6.1	0.0029	8.2	albumin	2.1	0.008	pvWMH	0.6	0.24

*including albumin and periventricular WMH volume. *FDR* false discovery rate, *lw* logworth, *p* p-value, *pvWMH* periventricular white matter hyperintensities.

**Fig. 5:**
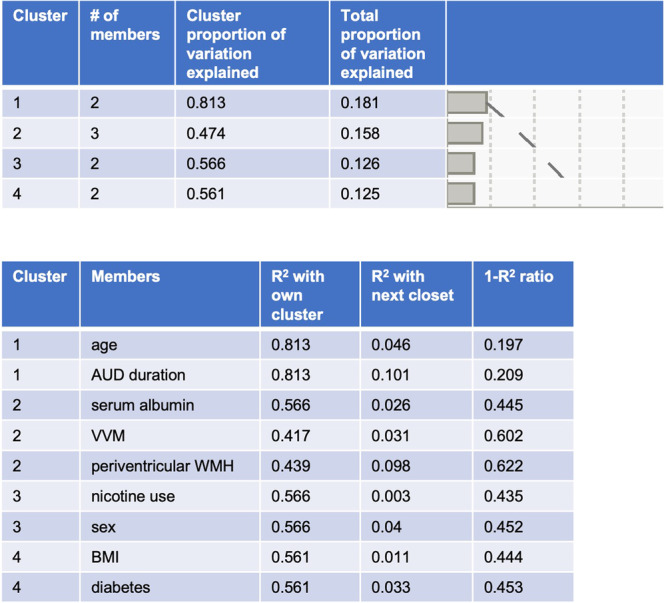
Result of a principal component analysis (PCA) including 9 variables associated with periventricular white matter hyperintensity (pvWMH) volumes the combined Alcohol Use Disorders (AUD) and HIV groups. AUD alcohol use disorder*,* BMI body mass index*,* VVM visual and verbal memory.

To confirm the salience of the identified relationships, a PCA was conducted in the combined HIV and AUD groups including the 9 variables that correlated with periventricular WM volumes (i.e., Fig. 2, 3: age, sex, albumin, BMI, nicotine use, hypertension, AUD duration; and visual and verbal memory performance). The PCA grouped the 9 variables into 4 clusters, the 2^nd^ of which included serum albumin, periventricular WMH volumes, and the visual and verbal memory Z-score (Fig. 5).

**Fig. 6:**
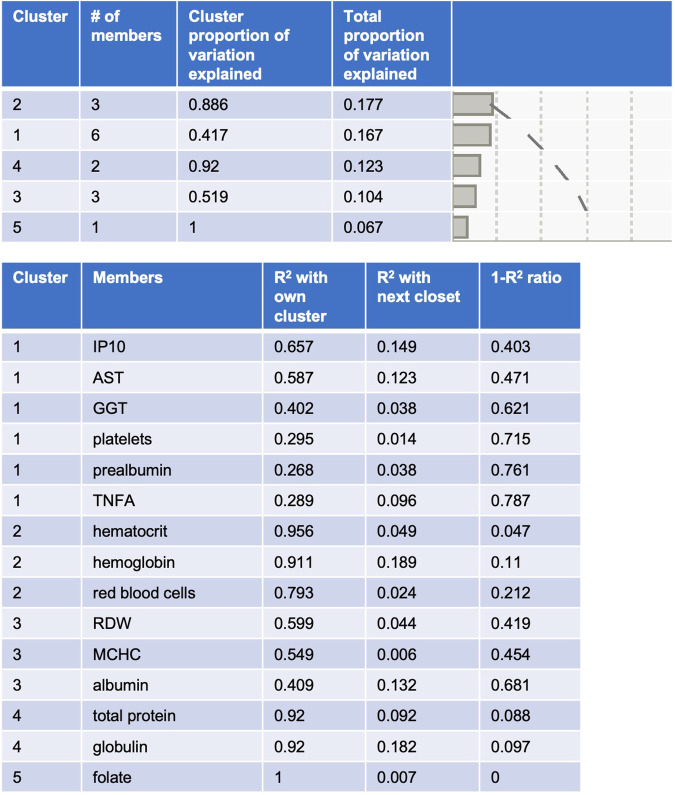
Result of a principal component analysis (PCA) including 14 blood markers associated with serum albumin levels or HCV. AST aspartate aminotransferase, GGT *γ*-glutamyl transferase, IP10 Interferon gamma-induced protein 10, MCHC mean corpuscular hemoglobin concentration, RDW red cell distribution width, TNFA tumor necrosis factor-α.

To help interpret the biological significance of low serum albumin levels, correlates with other blood markers were evaluated separately in the HIV and AUD groups. Blood marker differences as a function of HCV were also evaluated as a positive control for liver disease. Whereas correlates of serum albumin (i.e., hemoglobin, hematocrit, mean corpuscular hemoglobin concentration, and prealbumin) overlapped in the HIV and AUD groups, blood marker correlates of HCV infection were different and included higher levels of aspartate aminotransferase (AST) and *γ*-glutamyl transferase (GGT) (Table 3). A PCA including albumin and the 14 blood markers associated with albumin or HCV demonstrated that whereas AST, GGT, IP10, TNF, and prealbumin clustered together, serum albumin clustered with red cell distribution width and mean corpuscular hemoglobin concentration (Fig. 6).

**Table 3:** Relations between albumin levels or HCV serostatus and other blood markers.

	Albumin				HCV	
	HIV^a^ group		AUD^a^ group		HCV^ group	
	r	p	r	p	F	p
**Complete Blood Count (CBC)**						
White blood cell count (thousand/uL)	0.09	0.3132	−0.04	0.5896	0.29	0.5912
Red blood cell count (million/uL)	0.21	0.0133	0.27	**0.0007**	2.82	0.0954
Hemoglobin (g/dL)	0.41	**<0.0001**	0.36	**<0.0001**	0.15	0.7009
Hematocrit (%)	0.35	**<0.0001**	0.29	**0.0002**	0.06	0.8111
Mean corpuscular (MC) volume (fL)	0.16	0.0618	−0.04	0.6073	5.69	0.0186
MC hemoglobin (MCH, pg)	0.22	0.0092	0.06	0.4774	3.66	0.0580
MCH concentration (g/dL)	0.27	**0.0016**	0.22	**0.0050**	0.60	0.4415
Red cell distribution width (%)	−0.27	**0.0010**	−0.18	0.0283	5.12	0.0253
Platelet count (thousand/uL)	−0.06	0.5035	−0.02	0.7847	16.75	**<0.0001**
Mean platelet volume (fL)	0.18	0.0786	−0.17	0.0813	1.56	0.2145
**Metabolic** **Panel**						
Protein (g/dL)	0.10	0.2262	0.42	**<0.0001**	30.26	**<0.0001**
Globulin (g/dL)	−0.47	**<0.0001**	−0.19	0.0170	69.53	**<0.0001**
Bilirubin (mg/dL)	0.09	0.2919	0.09	0.2629	0.04	0.8347
Alkaline phosphatase (U/L)	−0.07	0.4257	−0.04	0.6568	3.63	0.0593
Aspartate aminotransferase (AST, U/L)	−0.19	0.0251	−0.14	0.0854	26.09	**<0.0001**
Alanine aminotransferase (ALT, U/L)	−0.14	0.0988	0.03	0.7348	8.24	0.0045
*γ*-glutamyl transferase (GGT, U/L)	−0.05	0.5537	−0.11	0.1567	9.76	**0.0021**
Prealbumin	0.45	**<0.0001**	0.31	**<0.0001**	29.04	**<0.0001**
Blood urea nitrogen (BUN)	0.07	0.6330	0.08	0.4152	0.52	0.4722
Creatinine	0.15	0.2682	0.07	0.4810	0.34	0.5626
eGFR	−0.22	0.2097	0.15	0.2341	0.59	0.4468
**Nutrition**						
Vitamin B9 (folate)	−0.11	0.2778	0.22	**0.0055**	0.30	0.5845
Viamin B12 (cobalamin)	−0.05	0.5842	0.07	0.4167	4.61	0.0340
**Inflammation**						
TNFα	−0.07	0.6427	−0.15	0.1632	13.76	**0.0006**
IP10	−0.30	0.0382	−0.05	0.6467	56.66	**<0.0001**

^a^simple regressions; ^Welch’s test for unequal variances; eGFR=estimated glomerular filtration rate; Bold = signficant at Bonferroni-corrected *p*-value ≤ 0.005 (for 10 CBC markers), *p*-value ≤ 0.004 (for 12 metabolic markers), *p*-value ≤ 0.016 (for 3 nutrional markers); *p*-value ≤ 0.025 (for 2 inflammatory markers).

## Discussion

The current results demonstrate that low serum albumin levels contribute significantly to explaining variance in periventricular WMH volumes in HIV after consideration of other relevant factors. Further, low serum albumin levels contributed more than periventricular WMH volumes to worse performance on tests of visual and verbal memory in HIV. Finally, relations between serum albumin and blood markers suggest that in this sample, low serum albumin levels reflect hematological abnormalities. Both albuminuria and hypoalbuminemia predict cardiovascular event prevalence independent of traditional risk factors, and albuminuria has consistently been shown to correlate with WMH volumes [52–60]. The current report, however, is among the first to evaluate the serum marker for its contribution to WMH volume. Only 2 relevant previous reports were identified: assessment in those with systemic lupus erythematosus [68] determined an inverse relationship between serum albumin and WMH volume; in healthy older adults, however, this correlation did not emerge [69] (also see, [93]). Our results are consistent with these prior observations as the correlation was salient in the diagnostic groups but not in the control cohort.

Initial analyses herein confirmed statistically significant, inverse relationships between lower serum albumin levels and greater periventricular WMH volumes in both HIV and AUD groups (i.e., Fig. 1b). In multivariate analyses considering traditional cardiovascular-, study-, and disease- related variables, however, this relation persisted only in the HIV group (i.e., Figs. 2 and 3). The lack of an enduring relationship between serum albumin and periventricular WMH volumes after consideration of covariates in the AUD group may be because they had neither liver disease or low serum albumin levels. This finding comports with the literature as the current sample was screened for known liver disease, and extant data indicates that serum albumin levels are low only in AUD complicated by liver disease [6–8].

It remains unclear why periventricular and deep WMH volumes have different biological correlates, but the current study is not the first to reveal differential relations. Indeed, several studies have reported unique correlates of periventricular versus deep WMH volumes [94–96]. For example, in a sample of 155 dementia-free older adults, hypertension was associated with periventricular WMH, whereas diabetes correlated with deep WMH volumes [97]. That hypertension [94, 97] and a decline in total cerebral blood flow [98] can be related to larger periventricular but not deep WMH volumes invites the speculation that serum albumin may mediate larger periventricular WMH volume by effects on blood pressure or perfusion.

Several studies in both healthy and clinical populations have reported associations between cognitive functioning and serum albumin levels [61, 63–67, 99–106]; some specifically noting relations between lower serum albumin and deteriorated memory performance [107]. Indeed, HIV mono-infected [108] and HIV + HCV co-infected [109] individuals with cognitive impairment, including disturbed visual memory in HIV + HCV-coinfection [110], have low serum albumin. The current report, however, may be the first to establish a relationship between lower serum albumin and worse cognitive functioning in an HIV mono-infected cohort.

Differences in blood marker correlates observed between HCV and serum albumin levels in AUD and HIV suggest that albumin functions more than just an indicator of liver status. That is, whereas both HCV and serum albumin correlated with serum markers of liver function (i.e., prealbumin), only serum albumin additionally correlated with hematological markers (i.e., lower hemoglobin, lower hematocrit). These relations between serum albumin and altered hemodynamic profiles have previously been reported [111] and may reflect cardiovascular dysfunction [112–117].

A limitation of the current study was access to serum but not also urine albumin levels. Had urine albumin been available, the relationship between these two measures observed in other populations [21, 118–122] could have been evaluated; and more might have been inferred about the biological role albumin plays in contributing to greater periventricular WMH volumes.

In conclusion, the current study contributes to a nascent literature demonstrating relations between serum albumin and WMH burden in clinical populations including those with HIV. Determination of whether low serum albumin contributes mechanistically via effects on vasculature, blood pressure, or perfusion [123–125] to periventricular WMH volumes will require additional investigation.

### Supplementary information

Supplementary Table 1

## Data Availability

Data described in the manuscript, code book, and analytic code will be made publicly and freely available without restriction at https://data.mendeley.com/.
