# Supplementary material for: Serum albumin and white matter hyperintensities
Source: Transl Psychiatry. 2024 Jun 1;14:233. doi: 10.1038/s41398-024-02953-5 (PMC11144249; doi:10.1038/s41398-024-02953-5)
Supplement: Supplementary file 1 [file 41398_2024_2953_MOESM1_ESM.docx]

Table S1. Results of multiple regression models* for cognitive perfomance in the AUD						
	**model**		**R^2**	**principal source**		
	$F_{2,141}$	p		source	FDR lw	FDR p
executive functioning	6.4	**0.0021**	8.2	**pvWMH**	**2.6**	**0.003**
attention and working memory	7.7	**0.0007**	9.3	**pvWMH**	**2.7**	**0.002**
visual and verbal learning	5.3	0.0867	3.3	albumin	0.8	0.13
visual and verbal memory	3.2	0.0425	4.3	albumin	1.1	0.09
